# Dietary Patterns and Overweight/Obesity: A Review Article

**Published:** 2017-07

**Authors:** Min MU, Li-Fa XU, Dong HU, Jing WU, Ming-Jie BAI

**Affiliations:** Dept. of Preventive Medicine, School of Medicine, Anhui University of Science and Technology, Huainan, Anhui Province, China

**Keywords:** Dietary patterns, Overweight, Obesity, BMI, Meta-analysis

## Abstract

**Background:**

Dietary patterns analysis may provide insights into the influence of overall diet on overweight/obesity. In the past two decades, the relation between dietary patterns and overweight/obesity has been a research focus and a number of results were reported in the research field.

**Methods:**

An electronic literature search was conducted in PubMed and Web of Science, to identify human studies published by Mar 2015 and written in English. The following keywords or phrases were involved: dietary patterns, dietary pattern, factor analysis, principal component analysis, diet, obesity, adiposity, overweight and BMI. All the studies were retrieved and prudent/healthy (n=17) and western/unhealthy (n=18) dietary patterns were identified.

**Results:**

When compared with the lowest categories of a prudent/healthy dietary pattern, a reduced overweight/obesity risk was shown in the highest (OR=0.64; 95% CI: 0.52, 0.78; *P*<0.0001). While there was an increased overweight/obesity risk in the highest when compared with the lowest categories of a western/unhealthy dietary pattern (OR=1.65; 95% CI: 1.45, 1.87; *P*<0.0001).

**Conclusion:**

A prudent/healthy dietary pattern and limit intake of western/unhealthy dietary pattern should be followed, which helps to keep a healthy body mass.

## Introduction

In recent years, the prevalence of overweight/obesity has been increasing around the world (1). Resulting from an interaction of genotype and environment, overweight/obesity is a complicated multifactorial chronic disease (2). Therefore, the etiology of overweight/obesity needs to be understood and this is a condition associated with an increased risk of coronary heart disease, hypertension, diabetes mellitus, gallbladder disease, osteoarthritis and some types of cancers (3).

Currently, a mass of studies concerning diet and overweight/obesity have been published. However, the role of diet in the etiology of overweight/obesity remains controversial. One reason for inconsistent findings may be the traditional single nutrient-based approach in nutritional epidemiology, commonly applied in most nutritional epidemiological researches (4–5). Therefore, a measurement of the overall dietary intake-dietary pattern has been recommended, which reflects the dietary intake complexity as an approach to investigate the links between diet and disease (6).

A large number of studies employed factor analysis or principal component analysis to derive dietary pattern (7–23). In these statistical techniques, variables are aggregated into factors that represent the eating patterns of the population being studied. Among multiethnic women including Japanese and Chinese women (mean age of 53.9 yr for all subjects), the ‘meat’ pattern was positively associated with body mass index (BMI), whereas ‘vegetable’, ‘bean’ and ‘cold foods’ patterns were negatively correlated (24). Food factors could not consistently predict changes in BMI or obesity development (25), whereas dietary patterns were significantly related to BMI changes over time (7–23).

As various studies concerning dietary patterns and overweight/obesity have been published, the objective of this systematic review was to appraise critically the literature published to date and conduct meta-analysis to pool studies results, to clarify the association between dietary patterns and overweight/obesity.

## Methods

### Search strategies

An electronic literature search was conducted in PubMed and Web of Science, to identify human studies published by 2016 and written in English, included following keywords or phrases: dietary patterns, dietary pattern, factor analysis, principal component analysis, diet, obesity, adiposity, overweight and BMI. In order to identify studies that examined diet and overweight/obesity risk, four independent reviewers read the abstracts of articles acquired in the initial search. All reviewers agreed on relevant articles and full-text versions of articles were reviewed to identify studies that examined food and/or dietary patterns through the application of factor analysis, RRR and principal component analysis.

### Studies included criteria

To be eligible, a study had to fulfill the following criteria: first, it had to be an original report regarding the relationship between dietary patterns and overweight/obesity; and then, odds ratios (ORs) for overweight/obesity had to be presented (or the data to calculate them).

In order to reduce error, only the most common patterns of dietary consumption were identified from the remaining articles. Besides, selected dietary patterns were similar in terms of factor loadings of foods most commonly consumed within those dietary patterns. For example, the identified prudent/healthy dietary pattern tended to have high loadings of following foods, such as fruit, vegetables, poultry, fish, low-fat dairy and whole grains, while the western/unhealthy dietary pattern tended to have high loadings of following foods, such as red and/ or processed meats, refined grains, potatoes, sweets and high-fat dairy. Studies that identified dietary patterns having similar foods loadings with prudent/healthy and western/unhealthy patterns but were named differently were also included.

### Quality assessment

The Newcastle-Ottawa quality assessment scale was used for quality assessment. Ten questions were assessed and each satisfactory answer received 1 point, resulting in a maximum score of 10. Only studies for which the majority of questions were deemed satisfactory (i.e. with a score of 6 or higher) were considered to be of high methodological quality.

### Heterogeneity assessment

Chi-square test was adopted to test the heterogeneity across studies. Besides, a random-effects model was employed to account for the possible heterogeneity between studies, which defaulted to a fixed-effects model in the absence of heterogeneity (26). A *P-*value of less than 0.05 was considered significant.

### Statistical analysis

The original studies reported dietary pattern results in terms of quintiles, quartiles or tertiles of dietary factor scores and overweight/obesity, BMI. Therefore, a meta-analysis was conducted to combine the results, in which the overweight/obesity risk was evaluated in the highest when compared with the lowest categories of prudent/healthy and western/unhealthy. In addition, Review Manager, version 5.0 (Nordic Cochrane Centre, Copenhagen, Denmark) was adopted to conduct statistical analysis. ORs were pooled for dichotomous outcomes from each study, means ± standard deviation were pooled for continuous variables from each study and 95% CI for each outcome was estimated to reflect point estimate uncertainty. In order to determine whether differences in age, sample size, study design and race affected conclusions, sensitivity analysis was performed. By inspecting the funnel plot and formal testing funnel plot asymmetry with Begg’s test (27), publication bias was assessed. These calculations were carried out by applying Stata/SE, ver. 10 (Stata Corp., College Station, TX, USA).

## Results

### Overview of studies included in the systematic review and meta-analysis

**Fig. 1:**
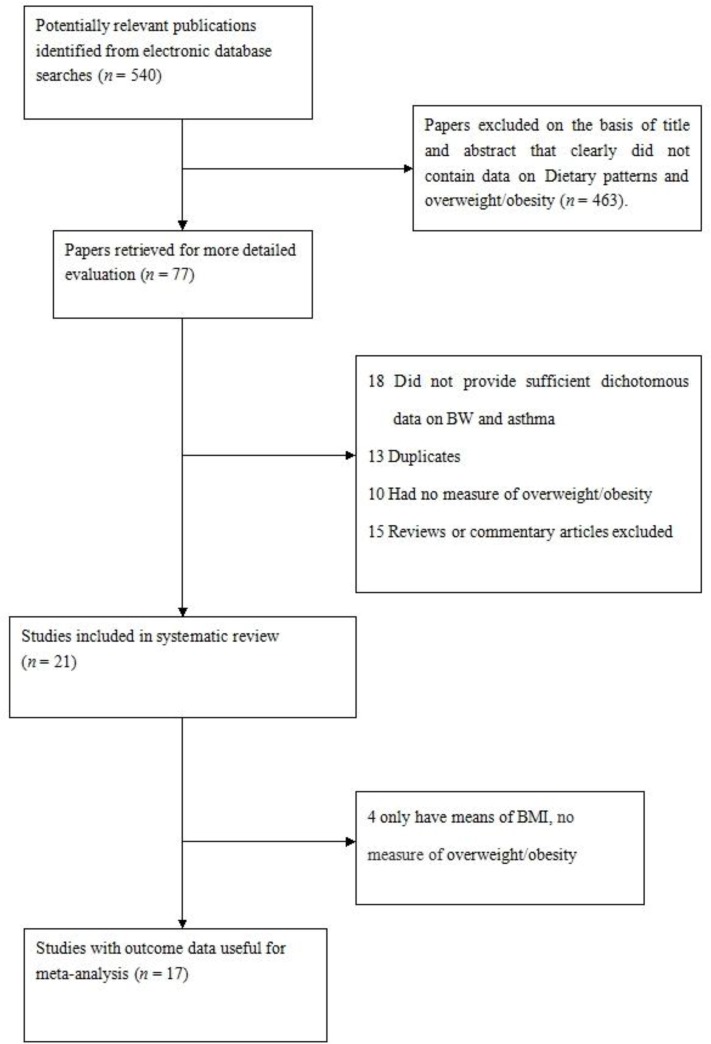
Flow chart of article screening and selection process

Fig. 1 showed the study selection process. Across the two databases, 540 papers fulfilled the search criteria. After reviewing the title and abstract, 463 articles were excluded, because the relationship between diet and overweight/obesity was not examined. Among the remaining 77 articles, 18 had dietary patterns but had no categorized participants by groups of dietary pattern scores, 13 were duplicates, 10 had no measurement of overweight/obesity, 15 were reviews or commentary articles, 4 only had means of BMI without measurement of overweight/obesity. Consequently, 17 articles including 18 studies (7–23) (one article (14) including 2 studies) met the inclusion criteria and they were included in the analysis. Study characteristics are displayed in Table 1.

**Table 1:** Characteristics of 17 articles (18 studies) included in the meta-analysis (1998–2015)

**Author Publication Year**	**Location**	**Study design**	**Total number of subjects**	**Race**	**Age**	**Definition of dietary pattern**	**Diet-assessment method**	**Dietary patterns identified**	**Outcome**
Pala V 2013	European countries	Cohort	14,989	White	2–10y	Principal component analysis	Children’s Eating Habits Questionnaire (CEHQ) (43; past one year)^1^	Snacking; Sweet and Fa; Vegetables and Whole meal, Protein and Water	overweight/obese
Okubo H 2008	Japan	Cross-Sectional	4, 394	Yellow	18–20	Factor analysis	Food-frequency questionnaire (30 groups; last month)^1^	Healthy; Japanese traditional; Western; Coffee and dairy products	Overweight; BMI
Paradis AM 2009	Canada	Cross-Sectional	664	White	18–55	Factor analysis	Food-frequency questionnaire (61; last month)^1^	Western; Prudent	Obesity; BMI
Nkondjock A 2010	Cameroon	Cross-Sectional	571	Black	21–59	Factor analysis	Food-frequency questionnaire (100; past one year)^1^	Fruits and Vegetables; Meats	Overweight and Obesity; BMI
Zhang JG 2105	China	Cohort	2,363	Yellow	18–44y	Factor analysis	24-h dietary recalls	Traditional south; Traditional north; Snack; High protein	Obesity
Hamer M 2009	UK	Cohort	2, 931	White	>16	Factor analysis	Interview(400; usual intake)^1^	Fast food; Health aware; Traditional; Sweet	BMI
Chan R 2014	China	Cross-Sectional	351	Yellow	10–12	Factor analysis	Food-frequency questionnaire (32; past one year)^1^	Fator 1; Fator 2; Fator 3;	Overweight and Obesity
Lioret S 2008	France	Cross-Sectional	748	White	3–11	Factor analysis	Interview (32; 7-d record)^1^	Pattern 1; Pattern 2	Overweight
Silva Bdel P 2014	Brazil	Cross-Sectional	1,026	White	20–60y	RRR	Food-frequency questionnaire (70; past one year)^1^	Fator 1; Fator 2; Fator 3;	Obesity
Suga wara N 2014	Japan	Cross-Sectional	338	Yellow	40.7	Principal component analysis	brief self-administered diet history questionnaire (BDHQ) (56 groups; past one year)^1^	Healthy; Processed Food ; Alcohol; Accompanying	Obesity
McDonald CM 2008	Colombia	Cross-Sectional	3, 075	White	5–12	Principal component analysis	Food-frequency questionnaire (38 items)^1^	Snacking; Cheaper protein; Traditional/starch; Animal protein	Overweight/Obesity
Shin KO 2007	Korea	Cohort	1, 441	Yellow	5.2	Factor analysis	Food-frequency questionnaire (100)^1^	Korean healthy; Animal foods; Sweets	Overweight
Denova-Gutierrez E 2011	Mexico	Cross-Sectional	6, 070	White	20–70	Factor analysis	Food-frequency questionnaire (116)^1^	Prudent; Westernized,; high animal protein/fat	Overweight/Obesity; BMI
Denova-Gutierrez E 2010	Mexico	Cross-Sectional	5, 240	White	20–70	Factor analysis	Food-frequency questionnaire (116; past one year)^1^	prudent, Western, and high protein/fat	Overweight/Obesity; BMI
Cho YA 2011	Korea	Cross-Sectional	1, 118	White	30–70	Factor analysis	Food-frequency questionnaire (103; past one year)^1^	Vegetable-Seafood; Meat-Fat; Snack	Overweight; Obesity

1 Number of food items and reference period in parentheses

### Meta-analysis

#### Prudent/healthy dietary pattern

**Fig. 2:**
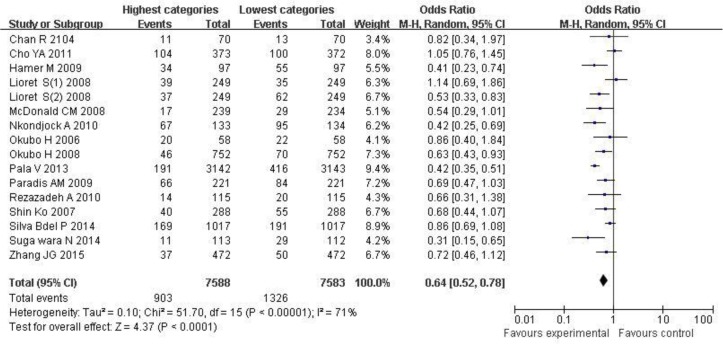
Forest plot for ORs of the highest compared with the lowest categories of intake of the prudent/healthy dietary pattern and overweight/obesity

When all studies were combined in the random-effects model, there was evidence of a decrease in overweight/obesity risk in the highest (Fig. 2) compared with the lowest categories of the prudent/healthy dietary pattern (OR=0.64; 95% CI: 0.52, 0.78; *P*<0.0001). In this study, the heterogeneity was very apparent (*P*<0.0001, I^2^ =71%).

#### Western/unhealthy dietary pattern

**Fig. 3:**
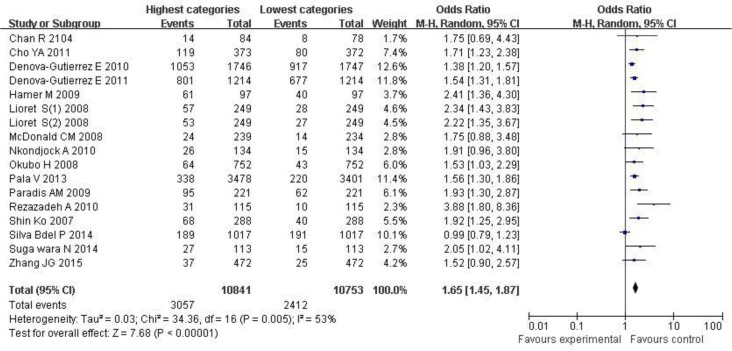
Forest plot for ORs of the highest compared with the lowest categories of intake of the western/unhealthy dietary pattern and overweight/obesity

Fig. 3 showed that when compared with the lowest categories (OR=1.65; 95% CI: 1.45, 1.87; *P*<0.0001), overweight/obesity risk would be increased in the highest categories in the random-effects model and there was less evidence of heterogeneity (*P*=0.13, I^2^=53%).

### Publication bias

Funnel plots revealed little evidence of asymmetry and thus little evidence of obvious publication bias. (The highest was compared with the lowest categories: prudent/healthy Begg’s test *P*=0.623; western/unhealthy Begg’s test *P*=0.070).

### Sensitivity analysis

Sensitivity analysis revealed that differences in age, sample size, study design and race had an effect on the association between dietary patterns and overweight/obesity.

When the highest were compared with the lowest categories of prudent/healthy dietary pattern, a negative association between prudent/healthy dietary pattern and overweight/obesity was more obvious (Table 2) in the following studies, which were of cross-sectional design (7–9,^14^,^17^,^23^) and in small sample size (7,9,10,14,21). Besides, subjects were white (7,9,12,14,17,23) and more than 20 yr old (8,14,17,18). In contrast, when compared with the lowest categories of western/unhealthy pattern (Table 2), a positive association was more obvious for those in the highest in studies that were of cohort design (12,18,21) and in small sample size (7,9,10,14,21,23). Furthermore, subjects were yellow and others (8,10,18,21) and less than 20 yr old (8,14,17,18). As these variables had a significant impact on the association between dietary patterns and overweight/obesity, their differences may partially explain the heterogeneity observed between studies.

**Table 2:** Dietary patterns and overweight/obesity: sensitivity analysis

**Study characteristic**	**Category**	**Prudent/healthy dietary pattern (95% CI)**	**Western/unhealthy dietary pattern (95% CI)**
Age (yr)	>20	0.58 (0.41, 0.81)	1.55 (1.42, 1.70)
	<20	0.80 (0.65, 0.98)	1.91 (1.54, 2.36)
Sample size	Large (>1000)	0.76 (0.50, 1.17)	1.51 (1.38, 1.65)
	Small (<1000)	0.59 (0.44, 0.80)	2.12 (1.74, 2.58)
Race	White	0.63 (0.45, 0.87)	1.59 (1.46, 1.74)
	Yellow and Other	0.71 (0.45, 1.13)	1.66 (1.31, 2.10)
Study design	Cross-Sectional	0.62 (0.48, 0.81)	1.59 (1.45, 1.73)
	Cohort	0.81 (0.36, 1.83)	1.84 (1.34, 2.51)

## Discussion

The results indicate that a prudent/healthy dietary pattern may decrease overweight/obesity risk, while a western/unhealthy dietary pattern may increase overweight/obesity risk. In this meta-analysis, the results from 17 articles including 18 studies (7–25) which published from 1998 to 2016 were evaluated. Diet in terms of food groups (e.g., fruit and vegetables) or its content of single nutrient (e.g., dietary fat or fiber) or single food could have been related to overweight/obesity, the relationships could not reflect between human diets complexity, high correlations between intakes of various nutrients or food items and various nutrient-to-nutrient biochemical interactions and overweight/obesity. Our findings facilitate to elucidate the potential relation between dietary patterns reflecting dietary intake complexity and overweight/obesity.

Sensitivity analysis revealed that age, sample size, study design and race had an effect on the association between dietary patterns and overweight/obesity, which may partially explain the evident heterogeneity between studies. More importantly, the cohort study has complete information regarding the subject’s exposure. Therefore, results of cohort studies were more convincing than cross-sectional studies in this review. When compared with a larger sample study, a smaller sample study could control the data quality better. However, a larger sample study would have higher credibility and stronger representativeness than a smaller sample. The race difference may result in different cooking methods or food groups, culturally related and may differ by ethnicity. In our studies, the relationships between dietary patterns and overweight/obesity were different in different races.

Relationships between dietary patterns and overweight/obesity are not a new topic because these associations have already been reported in previous studies (13,24). In a review of 30 cross-sectional studies, summarizing the associations between BMI and dietary patterns defined by means of diet index, factor or cluster analysis. Although dietary patterns of each study were identified by different statistical methods, a diet rich in fruits and vegetables was inversely associated with BMI and a diet rich in meat and fat was positively associated with BMI (28). In our analyses, when the results of all studies were pooled, both ORs and mean differences of BMI were all statistically significant when the highest was compared with the lowest categories of prudent/healthy and western/unhealthy dietary pattern, which made our review more credible.

Our meta-analyses may not include all studies. As some large studies were not involved in our meta-analyses, in which factor scores were not categorized. It should be taken into account that FFQ has limitations in judging dietary patterns. Nevertheless, this bias was also observed in a large number of nutritional surveys independent of the method adopted (28). Moreover, FFQ was shown to be a reproducible and valid tool to identify dietary patterns through factor analysis (29,30). Limitations and subjective nature of factor analysis and principal component analysis technique are widely acknowledged (31, 32), however, cannot be avoided. Therefore, any association shown in this analysis is likely to be attenuated due to these limitations.

In our analysis, some potential limitations should be considered. Firstly, significant heterogeneity was present in our analysis, which introduced a warning concerning the generalization of present results. Secondly, confounding factors were poorly handled in numerous selected studies. Consequently, data included in this meta-analysis may suffer from different degrees of completeness and accuracy. In order to compensate for data heterogeneity, a sensitivity analysis has been performed. Nevertheless, for a number of potential confounding variables, such as sex, physical activity, and economic status, a sensitivity analysis was unable to be performed through these potential confounding variables, because this information was not provided in original articles. These limitations must be noted and the results should be considered with caution.

In addition, other dietary patterns may have relation with overweight/obesity as well. For example, a Japanese traditional pattern was associated with increased overweight/obesity risk, while a coffee and dairy products pattern were related to decreased overweight/obesity risk (8). For the purpose of our analyses, only the most commonly identified dietary patterns were identified, which might, therefore, cause bias in our results.

## Conclusion

The results provide evidence of an inverse association between a prudent/healthy dietary pattern and overweight/obesity risk and a positive association between a western/unhealthy dietary pattern and overweight/obesity risk. Confirming this association may be significant for clinical implications, primary prevention strategies of overweight/obesity and overweight/obesity-related diseases. Our meta-analysis results probably highlight the association between dietary patterns and obesity/overweight risk.

## Ethical considerations

Ethical issues (Including plagiarism, informed consent, misconduct, data fabrication and/or falsification, double publication and/or submission, redundancy, etc.) have been completely observed by the authors.
